# The R3-MYB Gene *GhCPC* Negatively Regulates Cotton Fiber Elongation

**DOI:** 10.1371/journal.pone.0116272

**Published:** 2015-02-03

**Authors:** Bingliang Liu, Yichao Zhu, Tianzhen Zhang

**Affiliations:** National Key Laboratory of Crop Genetics and Germplasm Enhancement, Cotton Hybrid R & D Engineering Center (the Ministry of Education), Nanjing Agricultural University, Nanjing, China; National Key Laboratory of Crop Genetic Improvement, CHINA

## Abstract

Cotton (*Gossypium spp.*) fibers are single-cell trichomes that arise from the outer epidermal layer of seed coat. Here, we isolated a R3-MYB gene *GhCPC*, identified by cDNA microarray analysis. The only conserved R3 motif and different expression between TM-1 and fuzzless-lintless mutants suggested that it might be a negative regulator in fiber development. Transgenic evidence showed that *GhCPC* overexpression not only delayed fiber initiation but also led to significant decreases in fiber length. Interestingly, Yeast two-hybrid analysis revealed an interaction complex, in which *GhCPC* and *GhTTG1/4* separately interacted with *GhMYC1*. In transgenic plants, Q-PCR analysis showed that *GhHOX3* (*GL2*) and *GhRDL1* were significantly down regulated in −1–5 DPA ovules and fibers. In addition, Yeast one-hybrid analysis demonstrated that *GhMYC1* could bind to the E-box cis-elements and the promoter of *GhHOX3*. These results suggested that *GhHOX3* (*GL2*) might be downstream gene of the regulatory complex. Also, overexpression of *GhCPC* in tobacco led to differential loss of pigmentation. Taken together, the results suggested that *GhCPC* might negatively regulate cotton fiber initiation and early elongation by a potential CPC-MYC1-TTG1/4 complex. Although the fibers were shorter in transgenic cotton lines than in the wild type, no significant difference was detected in stem or leaf trichomes, even in cotton mutants (five naked seed or fuzzless), suggesting that fiber and trichome development might be regulated by two sets of genes sharing a similar model.

## Introduction

Trichomes are epidermal hairs found in most land plants. Only some cells are committed to form trichomes. The specificity of development and differentiation of the trichome cell makes it a perfect model to study the regulation of cell development and cell fate determination. Trichome development and root epidermal patterning have been studied in depth in the model plant Arabidopsis thaliana. In Arabidopsis, the R2R3-MYB protein GLABRA1 (GL1) [1], the basic helix–loop–helix proteins GLABRA3 (GL3) or ENHANCER OF GLABRA3 (EGL3) [2, 3] and the WD40 protein TRANSPARENT TESTA GLABRA1 (TTG1) [4] form a combinatorial regulatory complex MYB-bHLH-TTG1 to positively regulate trichome patterning. Furthermore, this complex regulates the downstream gene *GL2*, encoding a homeodomain (HD-Zip) protein required for normal trichome development [5]. In Arabidopsis, four homologous single MYB proteins, CAPRICE (CPC) [6], TRIPTYCHON (TRY) [7] and ENHANCER OF TRY and CPC1 and 2 (ETC1 and ETC2) [8, 9] have been identified as negative regulators of trichome initiation and patterning. These inhibitory proteins contain a DNA binding domain but no transcriptional activation domain. Protein interaction analysis in yeast has suggested that TRY or CPC can interrupt the functionality of the “activating” GL1-bHLH-TTG1 complex through competitive interaction with bHLH [3, 7, 10]. Therefore, these factors may work as negative transcriptional regulators [11]. Fortunately, the development of cotton fibers and Arabidopsis trichomes share a similar regulatory mechanism involving closely related transcription factors as well as a similar lateral inhibition signaling pathway [12], making these trichomes an excellent system for cotton fiber research.

Cotton (*Gossypium spp.*) fiber cells are seed trichomes derived from the epidermal layer of the cotton seed coat. The molecular components responsible for regulating fiber cell differentiation have not been fully characterized [13]. In cotton, transcription factors also play an important role in regulating fiber elongation. Overexpression of *GaMYB2*, which is homologous to *AtGL1*, rescues trichome formation in the gl1 mutant and induces the production of seed trichomes in Arabidopsis [14]. Moreover, constitutive overexpression of *GhMYB25* in transgenic tobacco results in an increase in branched long-stalked leaf trichomes [15]. Correspondingly, *GhMYB25*-silenced cotton exhibits alterations in the timing of rapid fiber elongation, resulting in short fibers, dramatic reductions in trichomes on other parts of the plant and reductions in seed production [16], while suppression of *GhMYB25*-like results in cotton plants with fiberless seeds [17]. An HD-Zip IV family transcription factor, *G. barbadense* Meristem Layer 1 (GbML1), interacts with *GhMYB25* and specifically binds to the L1 box of *GbRDL1* [18]. *GbPDF1* is involved in cotton fiber initiation through interacting with three proteins (PPIP1–PPIP3) [13]. Therefore, it appears that these transcription factors may require companion proteins to work with.

Here, we isolated *GhCPC* from *Gossypium* TM-1 0DPA ovules, which is homologous to *AtCPC* and encodes a negative regulator of cotton fiber elongation. Our work aimed to identity whether *GhCPC* could interact with other proteins to form a MYB-bHLH-WD40 complex that regulates fiber elongation. Our results showed that *GhCPC* could interact with a bHLH gene, *GhMYC1*, while *GhMYC1* also interacts with *GhTTG1* and *GhTTG4*. In 35S::CPC transgenic lines, the levels of transcripts of the downstream genes *GhHOX3* and *GhRDL1* were significantly reduced. Using the yeast one-hybrid system, we found that *GhMYC1* could bind the promoter of *GhHOX3*. These results indicate that cotton fiber elongation may be regulated by a similar regulatory complex to that of Arabidopsis trichome development.

## Materials and Methods

### Plant Materials

The cotton lines used in this study included TM-1, the fiberless or fuzzless-lintless mutants XZ142fls, MD17 and SL1-7-1, the naked seed or fuzzless-linted mutants N_1_N_1_ and n_2_n_2_, *Gossypium raimondii, G. herbaceum* var. *africanum*, (MD17×TM-1)F_2_, two RILs [(MD17×TM-1)F_5_ and (SL1-7-1×TM-1)F_5_ populations] and transgenic lines. All of these lines were cultivated in Nanjing, China. Transgenic tobacco lines were grown in a greenhouse at Nanjing Agricultural University. Ovules and fibers were collected for analysis at different developmental stages and immediately frozen in liquid nitrogen. The day of anthesis was defined as 0 DPA.

### Gene Clone and Sequence Analysis

Genomic DNA and total RNA were extracted as described [19, 20]. Specific primers (CPC-full-F/R, S1 Table) were designed by electronic cloning to amplify the full-length *GhCPC* genomic sequence and cDNA sequence from TM-1. The promoters of *GhCPC* and *GrCPC* were amplified by TAIL-PCR [21]. The PCR products were ligated to T-vector pMD19 (Takara, Dalian, China) and then sequenced using GenScript (Nanjing, China). Amino acid sequences from different plants species were chosen using Blast software from NCBI. Alignments of homologous peptide sequences were carried out with ClustalX software (version 1.81) [22] and a neighbor joining phylogenetic tree was constructed using MEGA 4.1 [23].

### Q-PCR analysis

Ovules and fibers were collected from different plants from the same lines at the same time and place with three biological replicates. Total RNA samples (2 μg per reaction) of different tissues were reverse transcribed into cDNAs using AMV reverse transcriptase. The expression of genes involved in this study was analyzed by quantitative real time-PCR (Q-PCR). Q-PCR assays were performed in a 7500 Real-Time PCR System (Applied Biosystems) using First Start Universal SYBR Green Master (Roche). The cotton *Histone3* gene (AF024716) was used as an internal control and the relative expression levels of the genes were calculated using the comparative threshold cycle method. The amplification efficiency of each gene was calculated. The Q-PCR cycles were as follows: (1) 95°C, 10 min; (2) 40 cycles of 95°C for 15 s, 60°C(temperature varied for different primers, S1 Table) for 30 s and 72°C for 30 s; (3) a melting curve analysis from 65 to 95°C (1 s hold per 0.2°C increase) to check the specificity of the amplified product. Relative expression levels were determined by the 2^－ΔCt^ method.

### Yeast two-hybrid assay

The yeast two-hybrid assay was performed using the Matchmaker GAL4 Two-Hybrid System following the manufacturer’s protocol (Clontech). Full-length cDNA of *GhCPC* was fused to the GAL4-DNA-binding domain of the bait vector pGBKT7 and transformed into yeast strain Y187. A cDNA library from cotton ovules and fibers (0–20 DPA) was constructed by transforming yeast strain AH109 with ds cDNA and the pGADT7-Rec vector according to the manufacturer’s instructions. The library host strain was mated with bait strain Y187, and the mating mixture was then spread onto SD/–His/–Leu/–Trp medium and incubated at 30°C for 3–4 days. The transformants were selected and the positive clones were isolated and retransformed to bait strains to test their interaction using pGBKT7-53/pGADT7 as the positive control and pGBKT7-Lam/pGADT7 as the negative control.

### Yeast one-hybrid assay

The yeast one-hybrid assay was performed using the MATCHMAKER onehybrid system (Clontech). The *HOX3* promoter region (−1,798–1, 229 bp) was amplified from the TM-1 genome and fragments of 4×E-box-WT and 4×E-box-Mutant were synthesized by GeneScript Bio-Technology Co., Ltd. These three fragments were ligated into the *HindIII*- *XhoI* sites of pAbAi. The bait constructs were linearized with *BstBI* and integrated into the yeast genome (strain Y1H). Various concentrations of Aureobasidin A (AbA; Clontech, Cat.NO.630446) on SD/-Ura medium were used to identify the basal expression of AUR1-C. The ORF of *GhMYC1* was ligated to the GAL4 activation domain in pGAD424. Yeast transformants were tested on SD/-Ura medium containing 50ng/ml AbA.

### Plasmid construction and plant transformation

Cotton transformation was carried out as described [24]. To construct the overexpression and antisense vectors, two pairs of primers containing *Xba*I and *Sma*I sites were used to amplify *GhCPC* and the PCR products were ligated into pBI121 vectors, respectively.

The constructs were introduced into *Agrobacterium* strain LBA4404 for transformation. Cotyledon and hypocotyl explants from *G. hirsutum* cv. W0 were transformed using *Agrobacterium*-mediated transformation and selections were performed on kanamycin sulfate-containing medium. After approximately 10 months of *in vitro* culture and selection, the putative transgenic plants were transferred to a greenhouse for further screening and analysis. Homozygosis of transgenic plants was determined by segregation analysis based on the presence or absence of the kanamycin selection marker NPTII using specific primers for PCR detection as described above [25]. Another primer set was used to determine the presence of the promoters and the *GhCPC* transgene.

The homozygous transgenic lines were developed by pedigree selection and further used to test fiber length of T3 generation plants in Nanjing in 2012. Fiber samples of transgenic lines and the wild type were tested at the Supervision, Inspection and Test Center of Cotton Quality, Ministry of Agriculture in China.

### Scanning electron microscopy of initial fibers at 0 DPA

To detect the differences in initial fibers between CPC-overexpression transgenic lines and the wild type, ovules were simultaneously collected from the same position and fixed in 3% glutaraldehyde. After washing, dehydration, incubation and drying, the cotton ovules were examined under an S-3000N SEM (Japan).

### Accession numbers

Sequence data of genes in this article are deposited in GenBank as follows: *GhCPC* (FJ402930), *GhHOX3* (AY626159), *GaRDL1* (AY641990), *GhTRY* (JN997398), *GhTRY* (JN997398), *GhTTG1* (JQ005876), *GhTTG2* (AF530910), *GhTTG3* (AF530911), *GhTTG4* (AF530912), *GhDEL61* (JN997401), *GhDEL65* (JN997400), *AtCPC* (819249), *AtTRY* (835401).

*GhETC1 and GhMYC1* have not been uploaded onto NCBI.

## Results

### Isolation and Characterization of *GhCPC* in Cotton

In a previous study, we detected an EST with significantly different expression levels between TM-1 and fuzzless-lintless cotton mutants. Here, we isolated the gene from TM-1 0 DPA ovules and designated it as *GhCPC* (FJ402930), which is homologous to *AtCPC*. Sequence analysis showed that this gene encoded the same amino acids as contig16590, but not as contig17149 (S1 Fig.).

**Figure 1:**
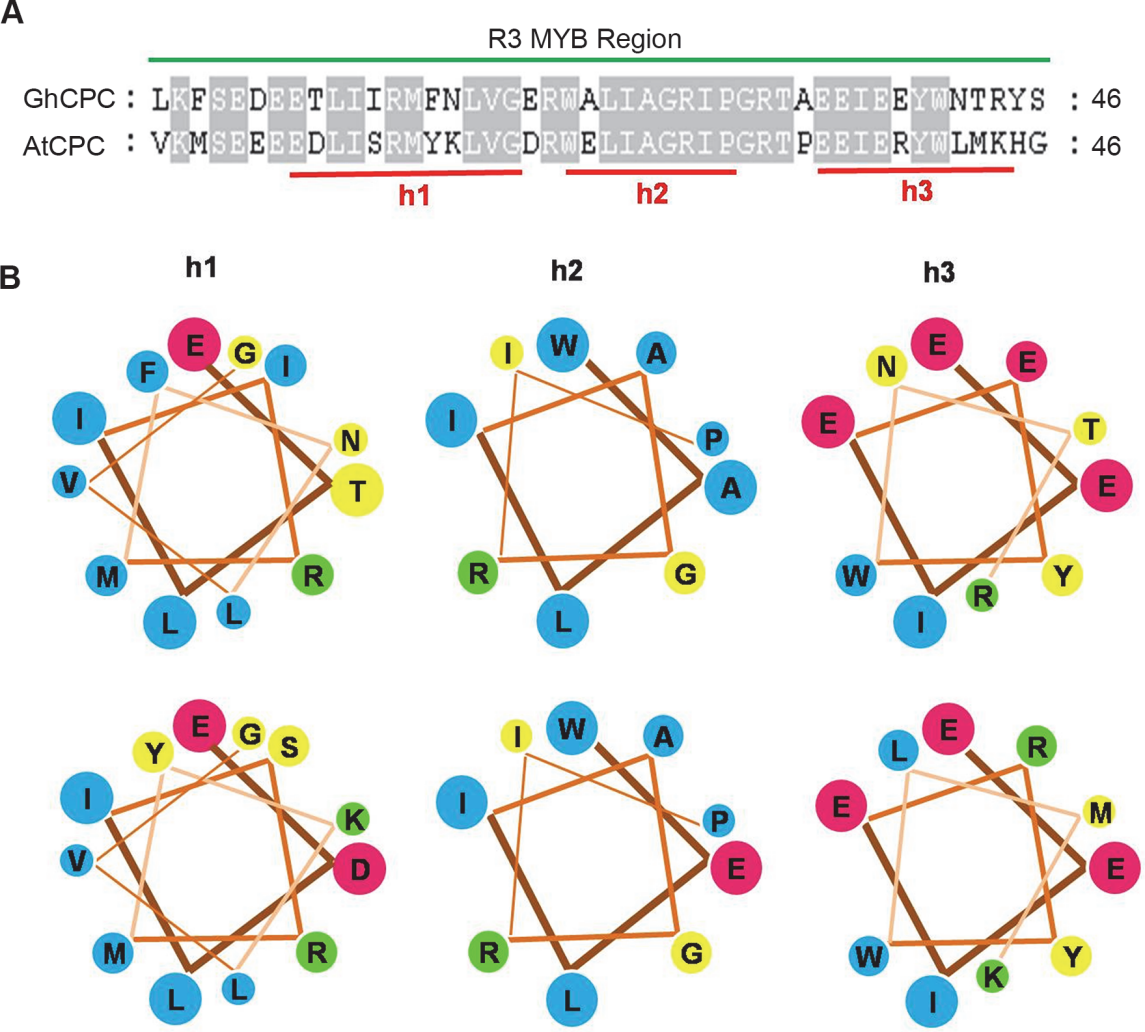
Protein comparison of *GhCPC* (R3 MYB) and *AtCPC* (R3 MYB). **A.** Sequence alignment of *GhCPC* and *AtCPC* R3 proteins. Shaded letters indicate identical residues. Green lines shows the positions of the three helices (h1, h2, h3) forming R3 MYB. **B**. Helical diagrams of h1, h2 and h3 in GhCPC R3 and AtCPC R3 with non polar residues in blue, polar residues in yellow, acidic residues in red and basic residues in green.

Both *GhCPC* and *AtCPC* encode a single R3 MYB repeat protein which shares 65% sequence identity. Like c-MYB, the R3 motif is composed of three helices (h1, h2, h3; Fig. 1A and 1B) [26, 27]. A comparison of the helices of *GhCPC* and *AtCPC* revealed that h1, h2 and h3 were similar. The high degree of homology between *GhCPC* and *AtCPC* suggests that these genes may have similar functions in controlling trichome or cotton fiber development through a potential MYB-bHLH-TTG1 regulatory complex.

We also identified CPC proteins in other species (*G. raimondii, Arabidopsis thaliana, Theobroma cacao, Vitis vinifera, Populus trichocarpa, Oryza sativa, Zea mays, Sorghum bicolor, Setaria italica, Panicum virgatum* and *Brachypodium distachyon*), but CPC proteins were only found in the eudicots, with none detected in monocots, according to genome annotation (http://phytozome.net/). These results suggest that CPC proteins did not differentiate along with the differentiation of eudicots and monocots. Sequence alignment revealed a domain conserved among different species that was critical for their function (S2 Fig.).

**Figure 2:**
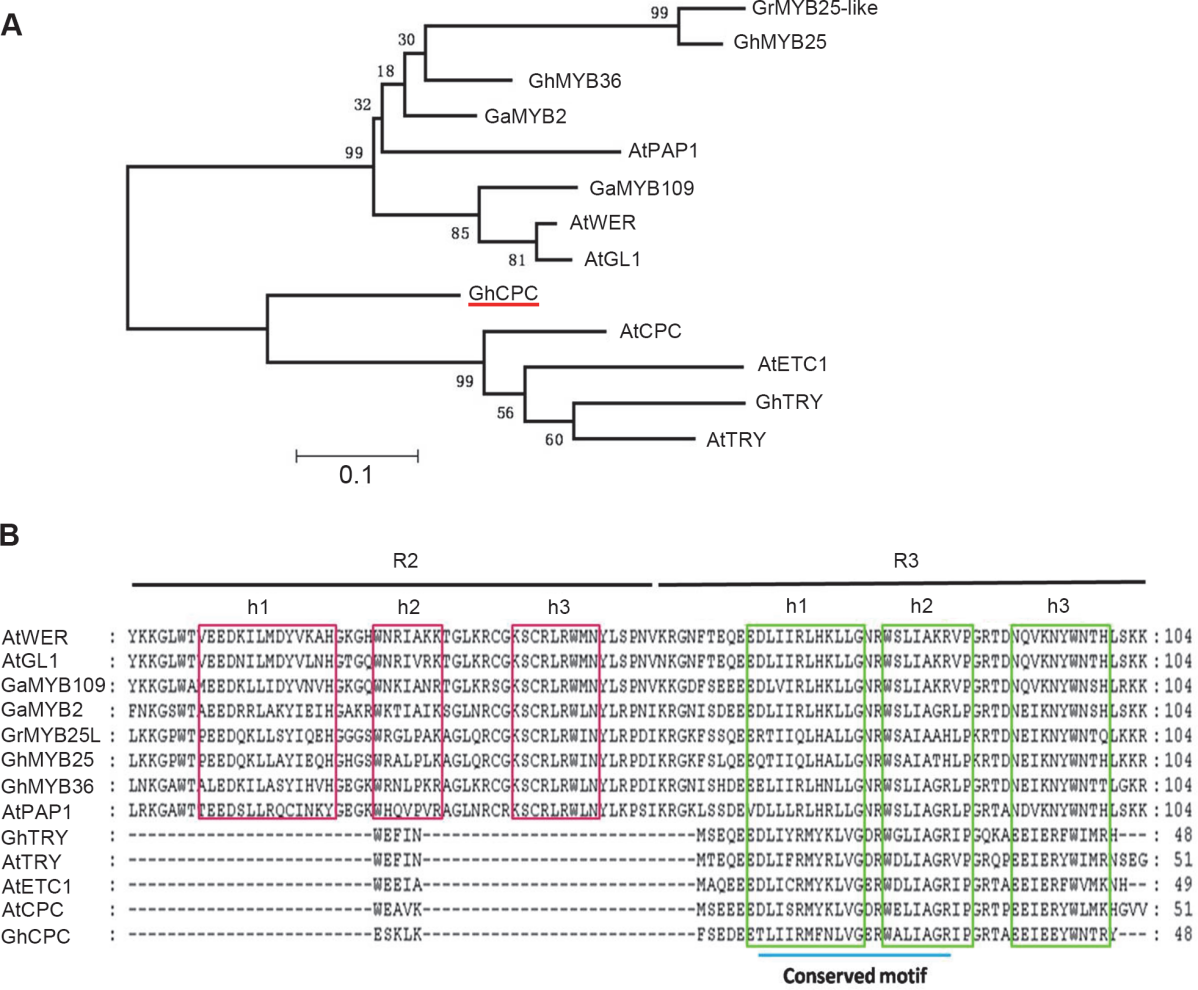
Phylogenetic tree and amino acid sequence alignment among the R2R3 MYB and R3 MYB regions. **A.** Neighbor joining phylogenetic tree of the amino acid sequence of the R2R3 MYB regions (*AtWER, AtGL1, AtPAP1, GhMYB2, GhMYB25, GhMYB25-like, GhMYB36, GhMYB109*) and R3 MYB regions (*AtETC1, AtTRY, AtCPC, GhTRY, GhCPC*). **B**. Sequence alignment of R2R3 MYB and R3 MYB members using ClustalX software. R2 and R3 domains are marked with black bars under the corresponding residues. Three helices of both the R2 and R3 domains are indicated with red and green boxes, respectively. The conserved MYB-bHLH interaction motif on the first and second helices of the R3 domain is underlined with a blue bar.

The MYB gene family is one of the most important transcription factor gene families in the plant kingdom. Comparative analysis of a gene family may reveal important adaptive changes at the protein level and thereby provide insights that relate structure to function. To provide a framework for identifying the evolution of R2R3 MYB and R3 MYB, a neighbor joining phylogenetic tree was constructed based on the R2R3 and R3 amino acid sequences (Fig. 2A). This tree has three distinct branches consisting of the genes *GhMYB25* (*GaMYB2, GhMYB25, GhMYB25-like, AtPAP1*), *GaMYB109* (*GaMYB109, AtWER, AtGL1*) and *GhCPC* (*GhCPC, GhTRY, AtETC1, AtCPC, AtTRY*; Fig. 2A). The full-length R2R3 MYB genes encode two DNA-binding domains, the R2 and R3 domain (Fig. 2B). Each domain has three conserved helices, and the structure of the third helix is essential for sequence-specific DNA binding. The first and second helixes of the R3 domain contain a conserved motif involved in MYB-bHLH interactions (Fig. 2B) [27–29]. As *AtCPC, GhCPC* also has only an R3 domain and exhibits a loss of DNA transcriptional activation of the R2 MYB domain. Previous studies showed *AtCPC* negatively regulated the trichome development by a MYB-bHLH-TTG1 complex [30, 31]. So we presumed that *GhCPC* might perform its function also as a negative regulatory gene in fiber development.

**Figure 3:**
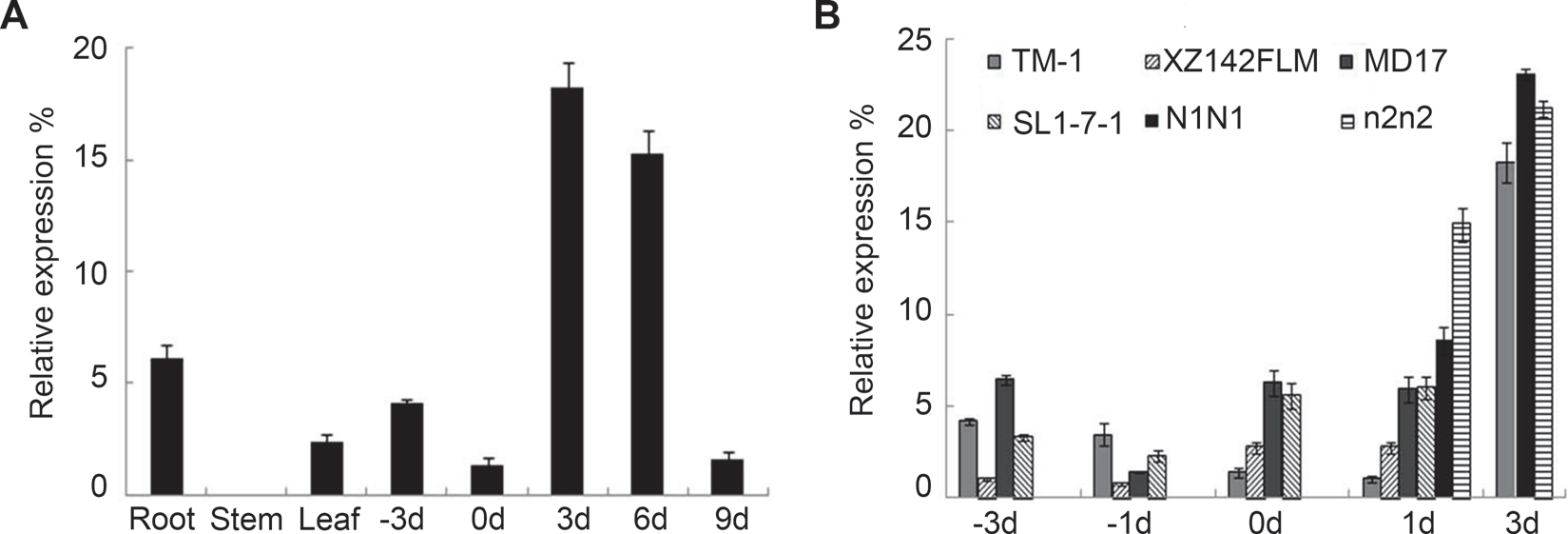
Q-PCR analysis of *GhCPC* expression in different cotton species. A. Spatial and temporal expression patterns in roots, stems, leaves, −3–0 DPA ovules and 3–9 DPA fibers in TM-1. B. Differential expression pattern in ovules of wild type (TM-1), three fiberless mutants (XZ142 FLM, MD17 and SL1-7-1) and two naked-seed mutants (N1N1 and n2n2).

To explore whether *GhCPC* functions as a negative transcriptional regulator of fiber initiation and elongation, we performed expression analysis of *GhCPC* in TM-1 and three fiberless or fuzzless-lintless mutants using quantitative real time-PCR (Q-PCR; Fig. 3). The results showed that the peak of *GhCPC* expression occurred in 3 DPA ovules during fiber elongation. The expression of *GhCPC* was at a lower level in leaves and roots, while no transcripts were detected in stems (Fig. 3A). Moreover, fuzzless-lintless (XZ142 fls, MD17, SL1-7-1) and fuzzless-linted or naked-seed (N_1_N_1_, n_2_n_2_) mutants had a significantly higher expression level than TM-1 in 0, 1 and 3 DPA ovules, while the opposite result was obtained in −1 DPA ovules (Fig. 3B). These results suggest that CPC may contribute to early fiber initiation and elongation and act as an inhibitor in the development of fuzzy seeds with lint.

**Figure 4:**
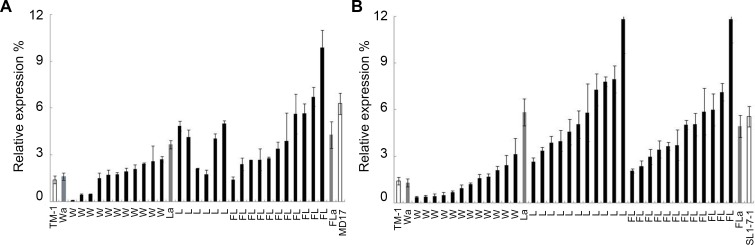
Q-PCR analysis of *GhCPC* expression in two RIL populations. **A, B** showed different expression pattern in 0–1 DPA ovules of pure lines of (MD17×TM-1) and (SL1-7-1×TM-1) RIL F_5_ population, respectively. W, L and FL on abscissa represent linted-fuzzy, linted-fuzzless and lintless-fuzzless lines, respectively. Wa, La and FLa on abscissa represent the average expression levels in the linted-fuzzy, linted-fuzzless and lintless-fuzzless lines, respectively.

To further examine the transcript levels of *GhCPC* in fuzzy-linted, fuzzless-linted and fuzzless-lintless cotton, we examined the expression of *GhCPC* among the three phenotypes in two RIL populations, (MD17 ×TM-1) and (SL1-7-1×TM-1) RILs(F_5_). First, we harvested 0–1 DPA ovules from 11 linted-fuzzy (wild type), six linted-fuzzless (La) and 11 lintless-fuzzless (FLa) lines derived from (MD17×TM-1) RILs (F_5_). The highest relative expression level of *GhCPC* was found in the fuzzless-lintless lines and the lowest was found in fuzzy-linted lines (Fig. 4A). Moreover, the average expression level was significantly lower in the wild type than in the La and FLa types, while it was lower in La than in FLa. However, the expression of *GhCPC* was lower in La and FLa than in MD17. In the other RIL population, *GhCPC* had a similar expression pattern in different lines. Specifically, we selected 12 wild type, 11 La and 12 FLa lines from (SL1-7-1×TM-1) RILs (F_5_). Similar to (MD17×TM-1) RILs (F_5_), *GhCPC* had a higher transcript levels in mutant type than wild type, while the highest transcript levels appeared in both the La and FLa lines (Fig. 4B). *GhCPC* expression was lower in FLa than in SL1-7-1, while the expression of this gene was also lower in the wild type than in TM-1. Though the wild type or lintless-fuzzless type lines had a similar phenotype to the corresponding parents, no identical expression level was detected. We presumed that the inhibition of *GhCPC* might be resulted from allele polymerization or genetic models and these also need further research to clarify.

### Overexpression of *GhCPC* Leads to Significant Decreases in fiber Length

To investigate whether overexpression of *GhCPC* would inhibit cotton fiber elongation, we generated CPC overexpression constructs driven by the 35S promoter and introduced these constructs into cotton (W0) *via Agrobacterium tumefaciens*-mediated transformation. Finally, we obtained 19 independent sense transgenic lines (T0), but only two antisense transgenic lines were obtained due to poor regeneration. We performed detailed phenotype and molecular analyses in the T3 generation plants. Compared to the wild type plants, which were separated from the T0 generation, overexpression of *GhCPC* resulted in shorter fibers in the *GhCPC* overexpression transgenic lines. However, no distinct difference was observed in the antisense transgenic lines, although the expression of *GhCPC* was lower in these lines than in the wild type (S3 Fig.), possibly because other genes, such as *GhTRY* or *GhETC1*, compensated for the loss of expression of *GhCPC* in the antisense lines.

**Figure 5:**
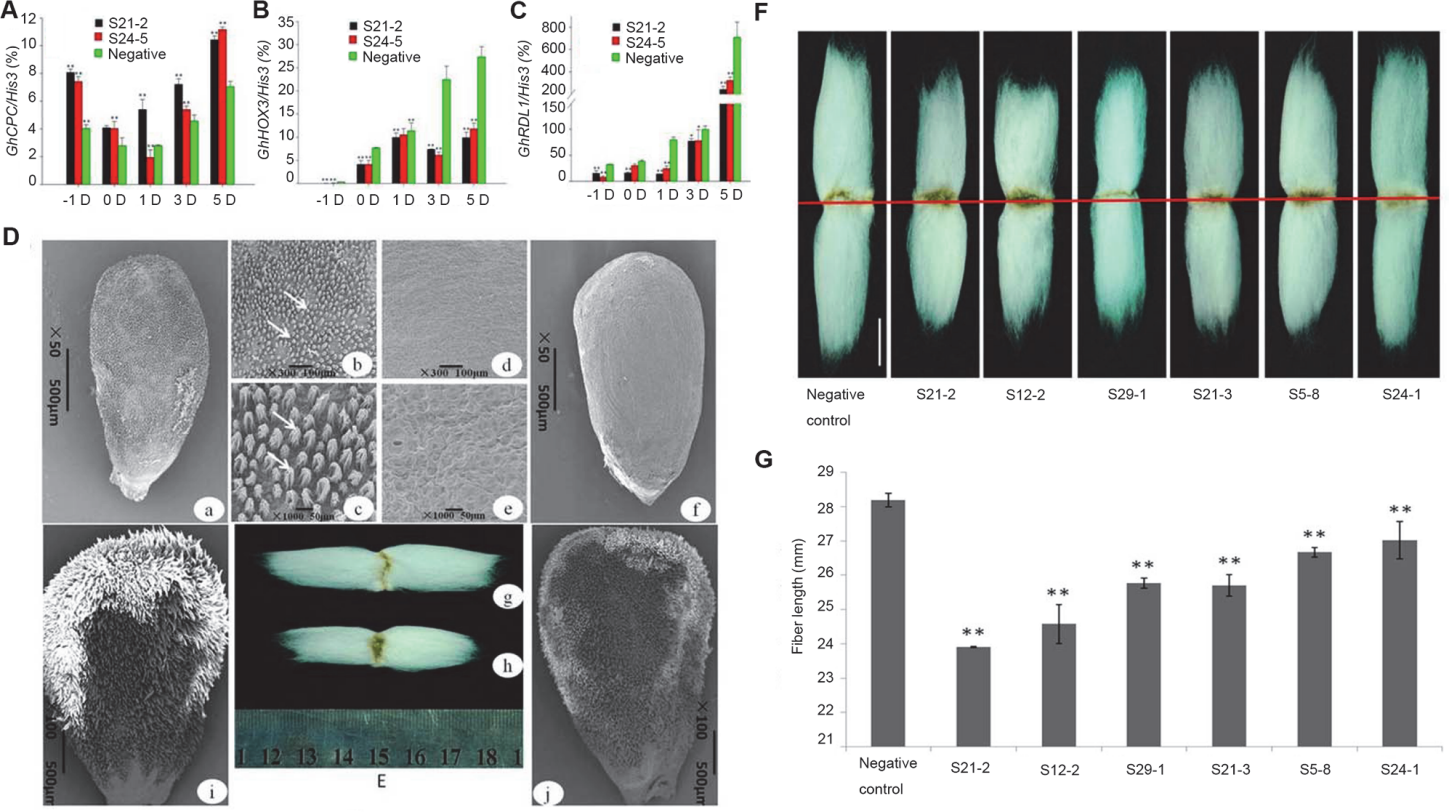
Compared to the wild type, overexpression of *GhCPC* leads to delayed fiber differention and decreased fiber length in the T3 generation Wild type was separated from the T0 generation. **A, B** and **C** respectively show significantly different expression of *GhCPC, GhHOX3* and *GhRDL1* in CPC sense transgenic lines S21-2 and wild type. **D**, Different initial development of fiber cells of 0 DPA between CPC-overexpression T3 S21-2 and wild type . **a, b, c** Wild type ovules exhibited normal differentiation and rapid emergence of fiber cells from the surface (**a**-**c**). However, CPC-overexpression ovules exhibited the opposite morphology in which the surfaces of ovules from the transgenic plants were smooth with no appearance of fiber initiation. The fiber cells were observed at 50×, 300× and 1,000× magnification. **E**, Mature fibers in the wild type (**g**) and S21-2 (**h**), respectively, corresponding to **D**-**a** and **D**-**f. F**, Mature fiber comparison among wild type and CPC overexpression lines. The white line represents 1 mm. **G**, Measurement of fiber length showed that the fiber length in transgenetic lines was shorter than that in the wild type. Bars represent SD of three measurements and ** represent p-value ≤ 0.01 (*t-*test).

In the *GhCPC* overexpression transgenic lines, we examined the expression level of *GhCPC* in −1, 0, 1, 3, 5-DPA ovules and fibers by Q-PCR. The result showed a similar expression trend that observed in TM-1. The accumulation of *GhCPC* transcripts was significantly higher in the ovules and fibers of the overexpression lines than in the wild type (Fig. 5A). Scanning electron microscopy (SEM) of the ovules of wild type plants revealed normal differentiation and rapid emergence of fiber cells from the surface of the ovule at 0 DPA. By contrast, the surfaces of the ovules from the transgenic plants S21-2 were smooth, with no appearance of fiber initiation (Fig. 5D). However, at 1 DPA, the fibers of *CPC*-overexpression plants began to develop, although their lengths were much shorter than those of the wild type. These results suggest that overexpression of *GhCPC* is first effective in fiber initiation. Integrating the regulatory model of Arabidopsis trichome development and the role of *GhHOX3* and *GhRDL1* in fiber elongation [14], we presumed that *GhHOX3* and *GhRDL1* are downstream genes of *GhCPC.* In the sense transgenic lines S21-2 and S29-1 (T3), Q-PCR showed that the transcript levels of *GhHOX3* were significantly reduced compared to the wild type, which was also true for *GhRDL1* (Fig. 5B–C). Mature fibers on S21-2 T3 homozygous transgenic plants were significantly shorter (23.90 ± 0.01 mm) than those of the wild type (28.14 ± 0.12 mm, *t*-test, *p*-value ≤ 0.01; Fig. 5E), while the other transgenic lines also exhibited shorter fibers to various degrees (Fig. 5F-G). These results suggest that CPC plays an important role in the early stages of fiber cell differentiation, and overexpression of CPC not only delayed initial fiber development, but it also repressed early fiber elongation. However, there were no significant changes in the trichome phenotype of leaves between CPC overexpression transgenic lines and the wild type,besides the number of trichomes between S21-2 (23.0 ± 1.7) and the wild type (23.4 ± 1.8) in the same field of view (20× stereomicroscope; S6 Fig.).

### Interaction of proteins of the CPC-MYC1-TTG1/4 regulatory complex by yeast two-hybrid assays

Previous studies have shown that *AtCPC* can interact with *AtGL3*, while *AtTTG1* also interacts with *AtGL3* [7, 32]. Examining similar interactions between *GhCPC* and other factors would improve our understanding of the role of *GhCPC* during cotton fiber differentiation. To identify whether *GhCPC* could interact with other proteins, the full-length *GhCPC* fused to the GAL4-DNA binding domain was used to screen a fiber yeast two-hybrid library using cDNAs from ovules and fibers from −3 DPA to 25 DPA. Two candidate interacting proteins were identified. One of the candidate proteins, *GhMYC1*, contained a MYC_N domain and a bHLH domain (S5 Fig.) and the other was a 60S ribosomal protein, named GhRibosomal, by homologous.

**Figure 6:**
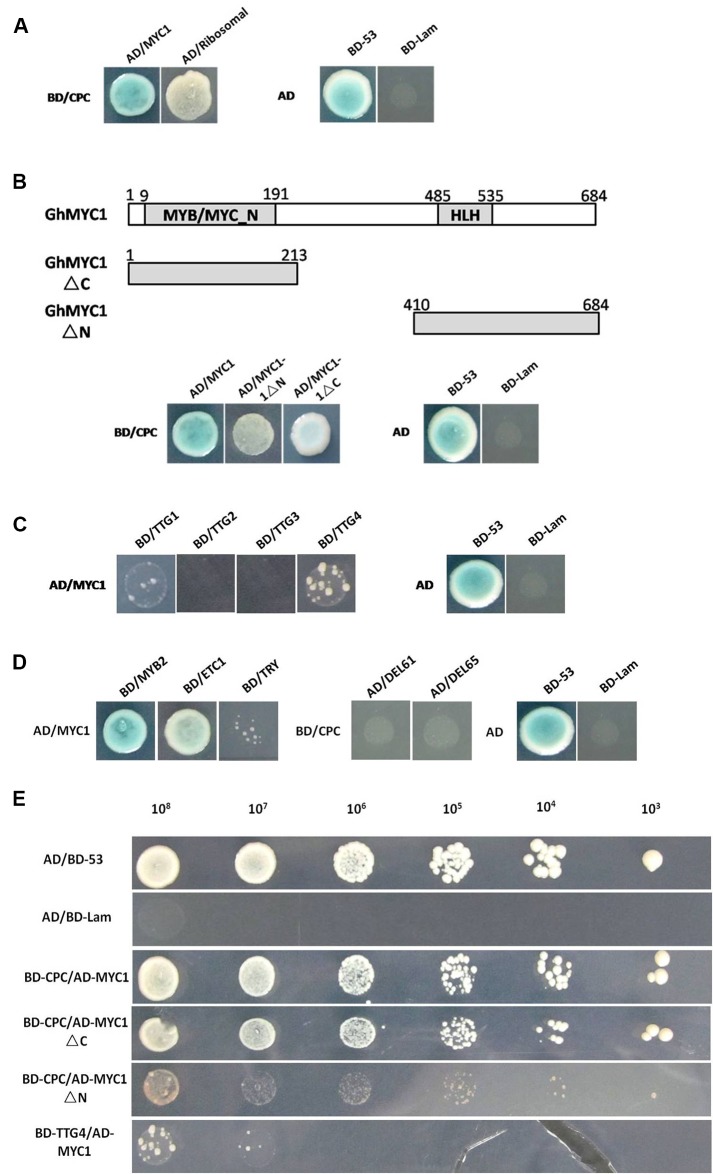
Interactions of different proteins. **A**. Yeast two-hybrid assays examining the interactions between CPC, MYC1 and ribosomal proteins. The vectors pGADT7/pGBKT-53 and pGADT7/pGBKT-Lam were separately used as positive and negitave controls. **B**. Mapping of trucated domains of MYC1 to bind to CPC. As shown, both the MYB/MYC domain and the bHLH domain are required for the interaction with CPC. **C**. Of the four WD40 proteins, only TTG1 and TTG4 had weak interactions with CPC. **D**. Interactions between different function genes. **E**. Different concentrations (cell/ml) were plated onto SD/-Ade-Leu-Trp-His medium to examine the intensity of the interaction in the positve control by the yeast two-hybrid assay.

Yeast co-transformed with *BD-GhCPC* and *AD-GhMYC1* or *AD-GhRibosomal* grew on SD/–Ade/–His/–Leu/–Trp selective medium and activated the reporter gene to produce a blue end product with X-α-Gal (Fig. 6A). Since *GhMYC1* contains two conserved motifs involved in MYC_N (aa 9–191) and bHLH (aa 485–535), the *GhMYC1* protein was divided into two fragments, the N terminus and the C terminus. Furthermore, using MYC1-truncated derivatives of the N terminus (deletion of C, ΔC) or C terminus (deletion of N, ΔN) fragments, we found that both MYC1ΔC and MYC1ΔN were sufficient to interact with CPC separately, while the interaction with MYC1ΔC was stronger than that of MYC1ΔN, but not as strong as that of the whole protein (Fig. 6B, E). These results suggested that the MYC1 amino terminal truncations differed in their ability to independently activate reporter gene transcription when fused to the AD, and both parts were together responsible for the interaction.

Like *GhMYC1*, both *GhDEL61* and *GhDEL65*, which are homologous to *AtGL3*, belong to the bHLH super family. Therefore, these two bHLH genes fused to the activation domain were separately co-transformed with *GhCPC*. Interestingly, no interactions were observed among the proteins (Fig. 6D), although protein sequence analysis showed that the conserved domains of the three bHLH proteins shared high identity (S6 Fig.).

The fact that *GhCPC* strongly interacted with *GhMYC1* suggests that there might be a regulatory complex consisting of CPC-MYC1-TTG in fiber development. Therefore, we examined whether *GhMYC1* could interact with the WD40 genes (*GhTTG1-4* homologous to *AtTTG1*) in cotton. Further study showed that *GhMYC1* could interact with *GhTTG1* and *GhTTG4*, but the interaction between them was not strong, while no interactions were observed with *GhTTG2* or *GhTTG3* (Fig. 6C); perhaps these genes may take part in different complexes in different pathways.

Together, these data demonstrate that the CPC-MYC1-TTG1/4 regulatory complex may play an important role in fiber elongation. Similar to Arabidopsis, the model for the role of this complex in cotton fiber development involves inhibition with a feedback mechanism. According to this model, *GhCPC* regulates fiber initiation and directs the production of the fibers by forming a non-activating complex, which does not promote the transcription of the downstream gene *GhHOX3.*

### MYC1 directly binds to *ProGhHOX3*

In the CPC sense transgenic lines, significantly decreased expression of *GhHOX3* was observed compared to the wild type. Previous *in vitro* and *in vivo* binding assays showed that the bHLH domain can bind to the G-box *cis*-element (CACGTG) and G-box-like motifs (AACGTG or CATGTG) [33–35]. In a broader sense, E-box elements (CANNTG) served as binding sites of bHLH transcription factors [36]. Sequence analysis by PLACE (http://www.dna.affrc.go.jp/htdocs/PLACE/) revealed that the *GhHOX3* promoter (~2 kb) contained seven E-box *cis*-elements, one of which is a G-box *cis*-element. To determine whether MYC1 directly binds to the promoter of *GhHOX3*, we performed DNA-protein interaction analysis using the yeast one-hybrid system. Three DNA fragments were respectively inserted into the pAbAi vector. One contained part of the *ProGhHOX3* and the other two were 4×E-box-WT (four tandem repeats of CACGTG) and 4×E-box-Mutant (four tandem repeats of CATAGA).

**Figure 7:**
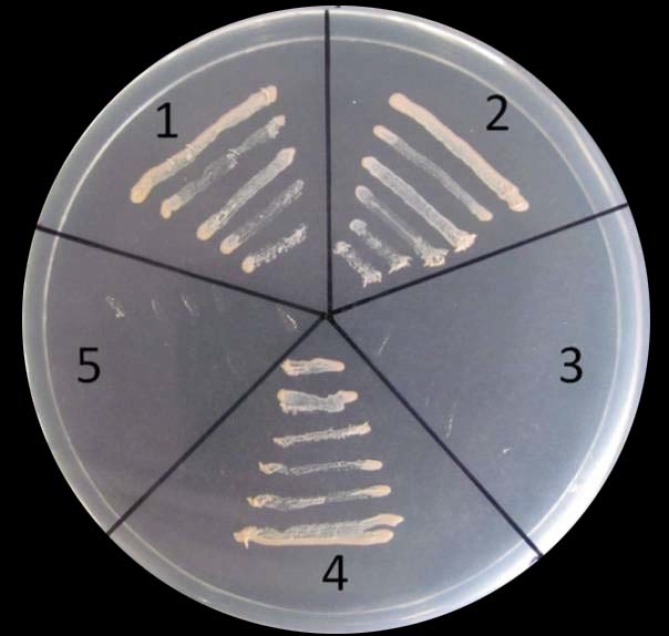
Transcriptional activation of ProHOX3, 4×E-box-WT and 4×E-box-Mutant by *GhMYC1.* (**1**) Yeast Y1H integrating ProHOX3, (**2**) 4×E-box-WT, (**3**) 4×E-box-Mutant or (**4**) p53-pAbAi were respectively transformed with *GhMYC1*.

Yeast one-hybrid analysis showed that *GhMYC1* could bind to the *ProGhHOX3* and 4×E-box-WT. However, when CACGTG was mutated to CATAGA, no binding activity to 4×E-box-Mutant was found (Fig. 7). These results indicate that the bHLH protein MYC1 is able to recognize and bind to the E-box motif and function as a transcription activator in yeast. The results also suggest that *GhMYC1* plays an important role in regulating the expression of *GhHOX3*. Also, like a bridge, when CPC binds to *GhMYC1*, they form a negative regulatory complex to inhibit downstream gene expression. If some positive regulators bind to *GhMYC1*, it may act as a promoter of fiber elongation.

## Discussion

### The potential role of *GhCPC* in cotton fiber elongation

Both cotton fibers and Arabidopsis trichomes are epidermal hairs, and they may share similar molecular mechanisms in the regulation of cell development. In Arabidopsis, *AtCPC* overexpression caused fewer trichomes than normal. Thus, the CPC gene determines the fate of epidermal cell differentiation in Arabidopsis [6]. In this study, *GhCPC* only had a conserved R3-domain with three helices and exhibits a loss of DNA transcriptional activation of the R2 MYB domain. *AtCPC* is a negative regulator of trichome initiation and patterning in Arabidopsis [6]. Since the R3 motif of *GhCPC* is homologous to *AtCPC* in terms of amino acid sequence, this protein may also have a similar function in fiber development. Expression analysis of *GhCPC* in TM-1, fiberless mutants and two RIL populations showed that *GhCPC* might serve as a negative regulator of fiber elongation. Previous studies had suggested that CPC played a critical role in determining the fate of epidermal cell differentiation in Arabidopsis [6]. We obtained a similar result in transgenic cotton plants. *GhCPC* overexpression led to significant decreases in fiber length, especially in 0 DPA ovules with no fiber protuberances, which is analogous to the dramatically reduced number of trichomes observed in *35S::CPC* and *35S::ETC1* transgenic Arabidopsis [6, 8]. This study showed that *GhCPC* delayed fiber elongation but did not completely inhibit cotton fiber early development, like *GhMYB25-like* silenced line [17]. These results reveal that *GhCPC* is a regulator of fiber elongation and that cotton fibers may share a similar model of cell differentiation with Arabidopsis trichomes.

### *GhCPC* functions through the potential formation of the CPC-MYC1-TTG1/4 regulatory complex

A network of three classes of proteins consisting of bHLH, MYB transcription factors and a WD40 repeat protein (TRANSPARENT TESTA GLABRA1, TTG1) act in concert to activate trichome initiation and patterning [11]. Evidence for the existence of the TTG1-bHLH-MYB complex is based entirely on protein interaction studies performed using the yeast two-hybrid system [2, 3, 28]. Protein interaction analysis has shown that *AtCPC* binds to the GL1-bHLH-TTG1 complex through competitive interactions with bHLH to inhibit trichome initiation when *AtCPC* is over expressed [6, 37, 38]. Our yeast two-hybrid data also show that *GhCPC* strongly interacted with a bHLH protein, *GhMYC1*, which is involved in gene regulation. *GhMYC1* could interact with *GhTTG1* and *GhTTG4* to produce the CPC-MYC1-TTG1/4 complex, which regulates fiber elongation in cotton.

In the regulatory complex, each partner may have similar or opposite genes on functions. In cotton, two bHLH proteins, *GhDEL61* and *GhDEL65*, are homologous to *AtGL1* and share 29% and 33% identity with *GhMYC1*, respectively. Amino sequence analysis among these three bHLH factors indicated that MYC_N motif of the N-terminal region and the HLH motif domain were highly conserved (S5 Fig.). Interestingly, there were no interactions detected between *GhCPC* and *GhDEL61* or *GhDEL65* in our yeast two-hybrid assays. Similarly, *GhMYC1* also had strong interactions with *GaMYB2* and *GhETC1* but weak interactions with *GhTRY*. Perhaps some different amino of conserved domains or non-conserved domains may block the interactions. The yeast two-hybrid assay results indicated that the proteins had distinct functions and evolutionary patterns or might have different favorite partner proteins. The results also suggested that different proteins might take part in different pathways to regulate different biological processes. Simultaneously, evidence showed that *GaMYB2* and *GhHOX3* had specific ways in which they cooperate to activate the *GhRDL1* promoter and for each pair of transcription factors, the effects seemed to be synergistic rather than additive [14]. Since *GhCPC* overexpression could decrease *GhRDL1* expression, we presumed that *GhCPC* and *GaMYB2* might have opposite effects on fiber elongation by competing to bind to *GhMYC1*.

**Figure 8:**
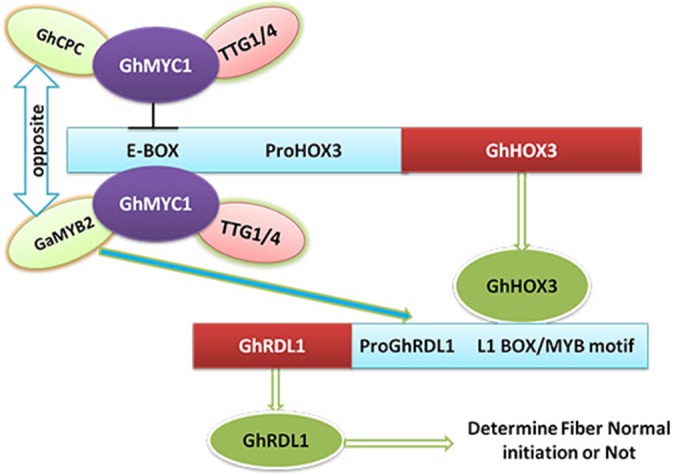
The potential CPC-MYC1-TTG1 regulatory complex in cotton. In this network, CPC-MYC1-TTG4 complex can regulate the expression levels of *GhHOX3* and *GhRDL1* by binding to their promoters. *GhCPC* and *GaMYB2* may have opposite effects on fiber development by completely binding to *GhMYC1*.

GLABRA2 (GL2) is a direct target of the GL1-GL3-TTG2 complex and is directly regulated by GL1 [39, 40]. In the current study, the levels of *GhHOX3* (homologous to *AtGL2*) transcripts in the transgenic lines were lower than those of the negative control. Since gel mobility shift experiments and yeast one-hybrid assays have previously shown that *AtCPC* does not bind to DNA [41], it is also unlikely that *GhCPC* represses *GhHOX3* by directly binding to its *cis*-elements sites to repress gene transcription. Similarly, in cotton, *GhCPC* may prevent the complex from binding to the promoter of *GL2*. In Arabidopsis, *AtMYC2* can directly target the E-box *cis*-element of the promoters of *TPS11* and *TPS21* [42], suggesting that the regulatory complex is indeed able to bind to the DNA fragments of the downstream genes. In the current study, the transcripts of *GhRDL1* were also significantly repressed by *GhCPC* overexpression in *35S::CPC* transgenic cotton lines. When *GhHOX3* genes driven by the 35S promoter was transferred into Arabidopsis, the *RDL-P3* promoter was activated to regulate GUS expression [14], while it could also transduce GA signal to promote fiber cell elongation [44]. Taken together, the results support a classical model in which *GhCPC, GhMYC1* and *GhTTG1*/*4* might form the CPC-MYC1-TTG1/4 complex to regulate the expression of *GhHOX3*, and subsequently, this GL2 protein regulates the downstream target gene *GhRDL1* to control fiber elongations (Fig. 8). Analysis of the bHLH domain of *GhMYC1* and the promoter of *GhHOX3* has shown that this conserved domain could recognize and bind to the E-box *cis*-element. Our yeast one-hybrid data confirmed this speculation.

In tobacco, flowers of *AtCPC* overexpression plants display an unexpected defect in pigmentation [43]. In light of the existence of the CPC-MYC1-TTG4 complex, *GhCPC* may have a similar function in regulating the production of anthocyanin. To further investigate the role of *GhCPC* and how conserved this role is across plant species, 35S::CPC was constitutively over expressed in tobacco (*Nicotiana tabacum*). In the transgenic lines, alterations in petal color also led to a differential loss of pigmentation (S7 Fig.). The results also indicate that *GhCPC* is involved in regulating anthocyanin biosynthesis, also possibly via the MYB-bHLH-WD40 complex. Further direct evidence of the function of this complex in regulating anthocyanin biosynthesis should be obtained in future studies.

### Differences in the trichome/fiber regulatory mechanism between cotton and Arabidopsis

Elucidating the regulatory pathway of *GhCPC* opens up new avenues for understanding the existence and importance of the CPC-MYC1-TTG1/4 complex in regulating fiber elongation and raises many questions about this regulatory network. Although they are both unicellular epidermal hairs, cotton fibers are distinct from Arabidopsis trichomes, while cotton fibers are produced in the seed and are unbranched and extremely elongated [45]. In this study, we also found that the regulatory system of cotton is not entirely identical to that of Arabidopsis. *GhCPC* overexpression produces seeds with shorter fibers in cotton, but no significant changes were found in leaf or stem trichomes, besides changes in the fibers of transgenic cotton and fiberless mutants (XZ142 FLM and MD17 FLM). We also found that there was no tissue-specific expression pattern for *GhCPC*. The silenced *GhMYB25-like* transcripts cotton produced fiberless seeds, but normal trichomes elsewhere [17], whereas *GhHD-1* silenced cottons were almost completely glabrous (hairless) and showed a delay but later normal seed fiber initiation [46]. Therefore, we hypothesize that the development of cotton fibers and leaf (or stem) trichomes is regulated by a similar model, but some genes suited for cotton fibers but not for leaf (or stem) trichomes. Here, we expounded the potential mechanism of *GhCPC* in regulating fiber development, although the findings of this study are similar to the Arabidopsis model, but the development of cotton fibers is much more complex than that of Arabidopsis trichomes. Compared to the complex and enormous regulatory network, short of enough evidences blocked the confirmation of the complex in fiber development. Elucidating the differences between these systems may further explain the specificity of the molecular regulatory mechanism of plant trichome development.

## Supporting Information

S1 Fig Sequence alignment of *GhCPC* homologous genes. (TIF)

S2 Fig Conserved CPC domain in different species. (TIF)

S3 Fig No difference in the fiber lengths between antisense transgenic lines and wild type. (TIF)

S4 Fig Trichome phenotypes of leaves and stems of *CPC* overexpression transgenic cotton and wild type. (TIF)

S5 Fig Nucleotide sequence and deduced amino acid sequence of full-length *GhMYC1* cDNA. (TIF)

S6 Fig Protein sequence alignment of *GhMYC1, GhDEL61* and *GhDEL65*. (TIF)

S7 Fig Clear phenotypic change in petal pigmentation in CPC overexpression T0 lines. (TIF)

S1 Table Oligonucleotides used in this study. (XLSX)
