# Supplementary figures and images for: The R3-MYB Gene GhCPC Negatively Regulates Cotton Fiber Elongation
Source: PLoS One. 2015 Feb 3;10(2):e0116272. doi: 10.1371/journal.pone.0116272 (PMC4315419; doi:10.1371/journal.pone.0116272)

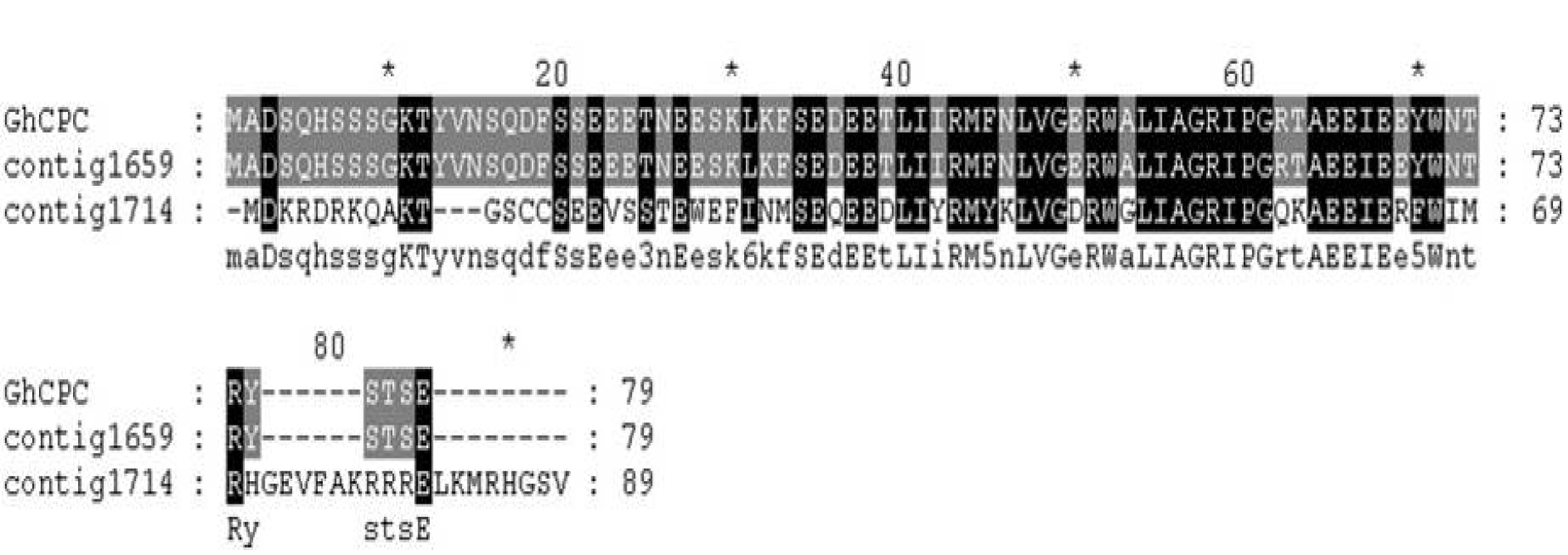

Supplement: S1 Fig — (TIF) [file pone.0116272.s001.tif]

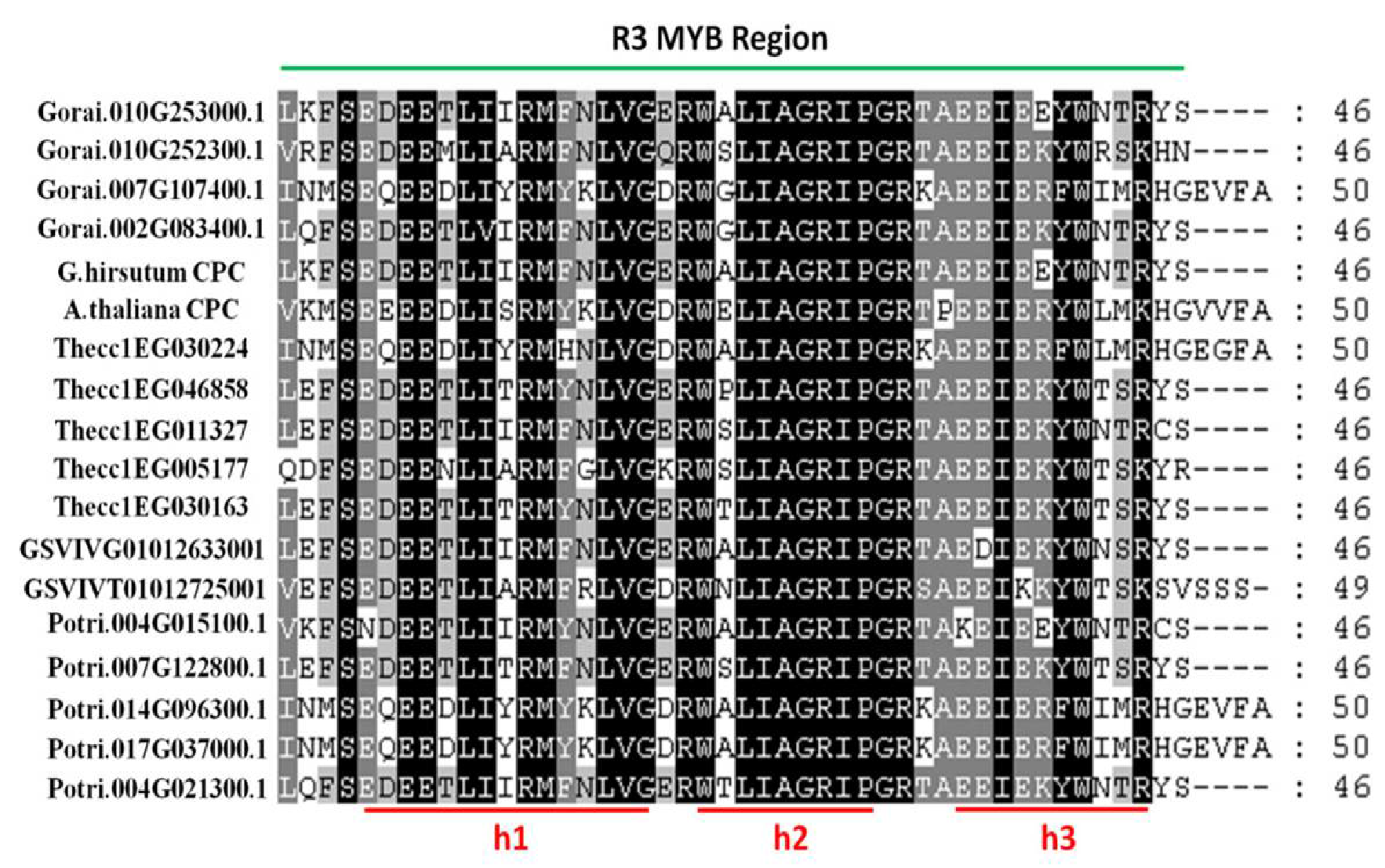

Supplement: S2 Fig — (TIF) [file pone.0116272.s002.tif]

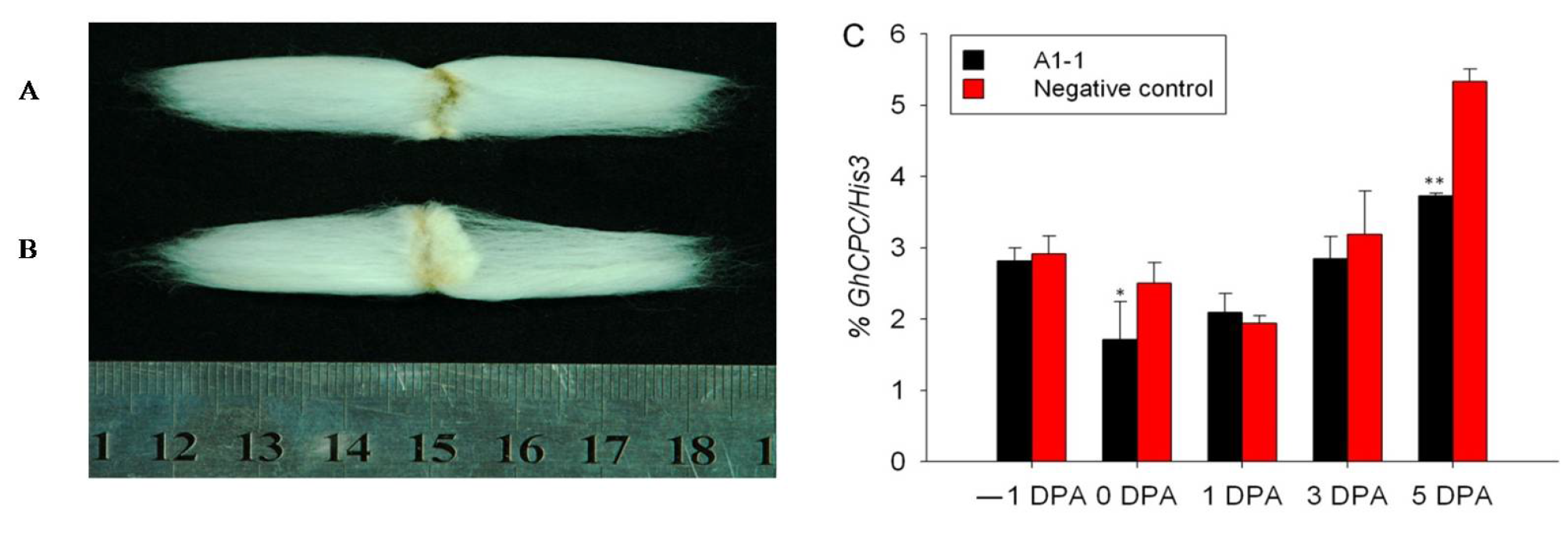

Supplement: S3 Fig — (TIF) [file pone.0116272.s003.tif]

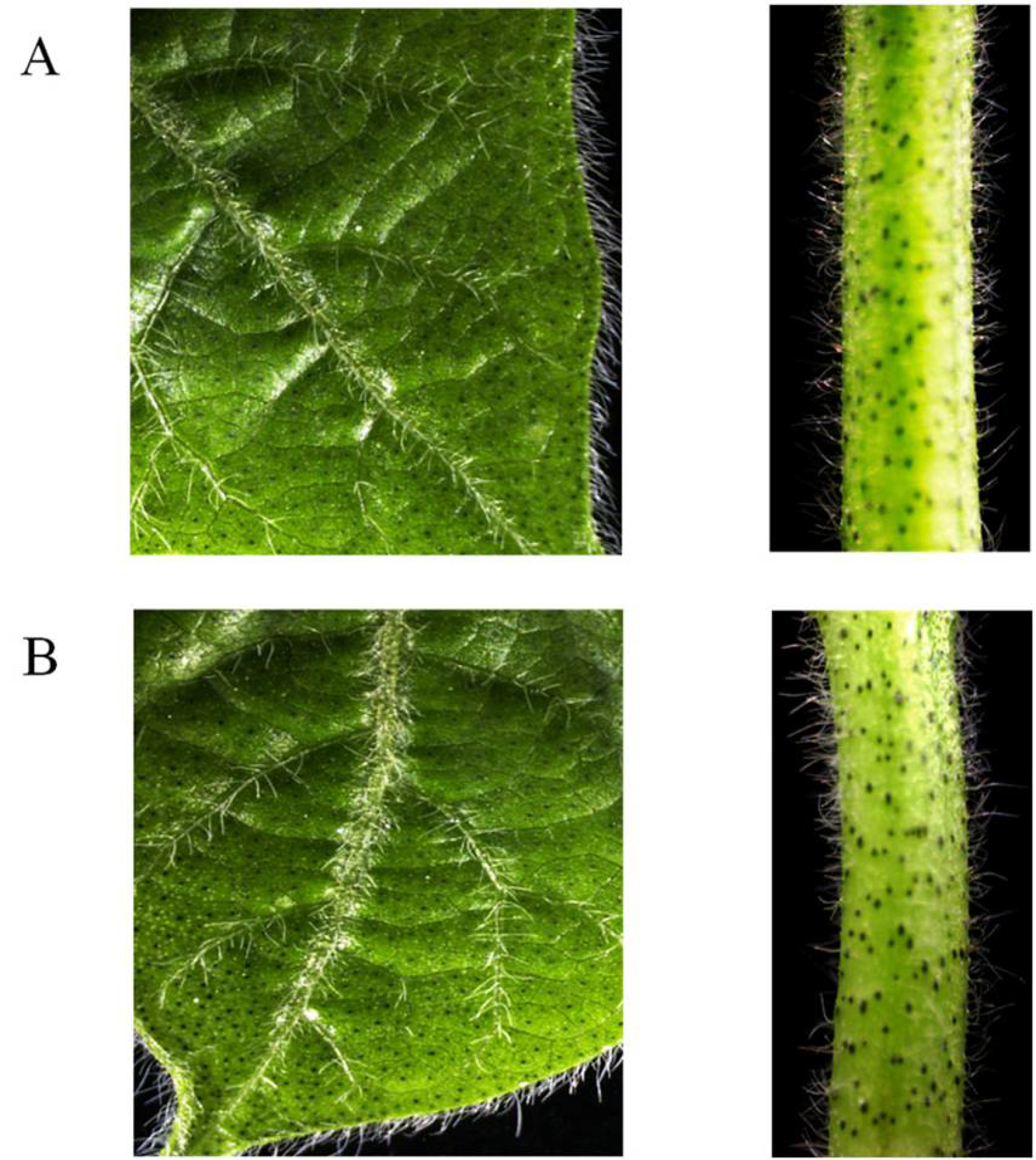

Supplement: S4 Fig — (TIF) [file pone.0116272.s004.tif]

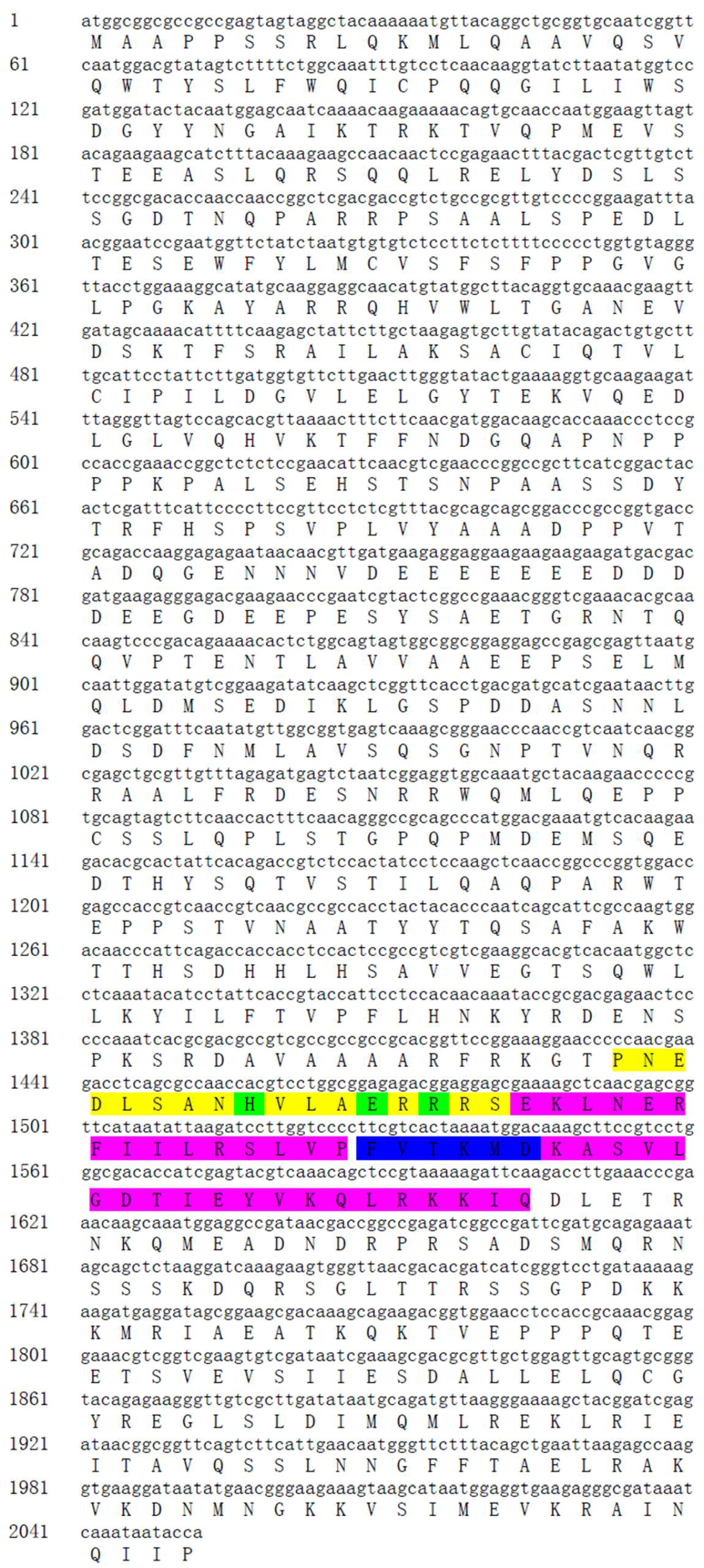

Supplement: S5 Fig — (TIF) [file pone.0116272.s005.tif]

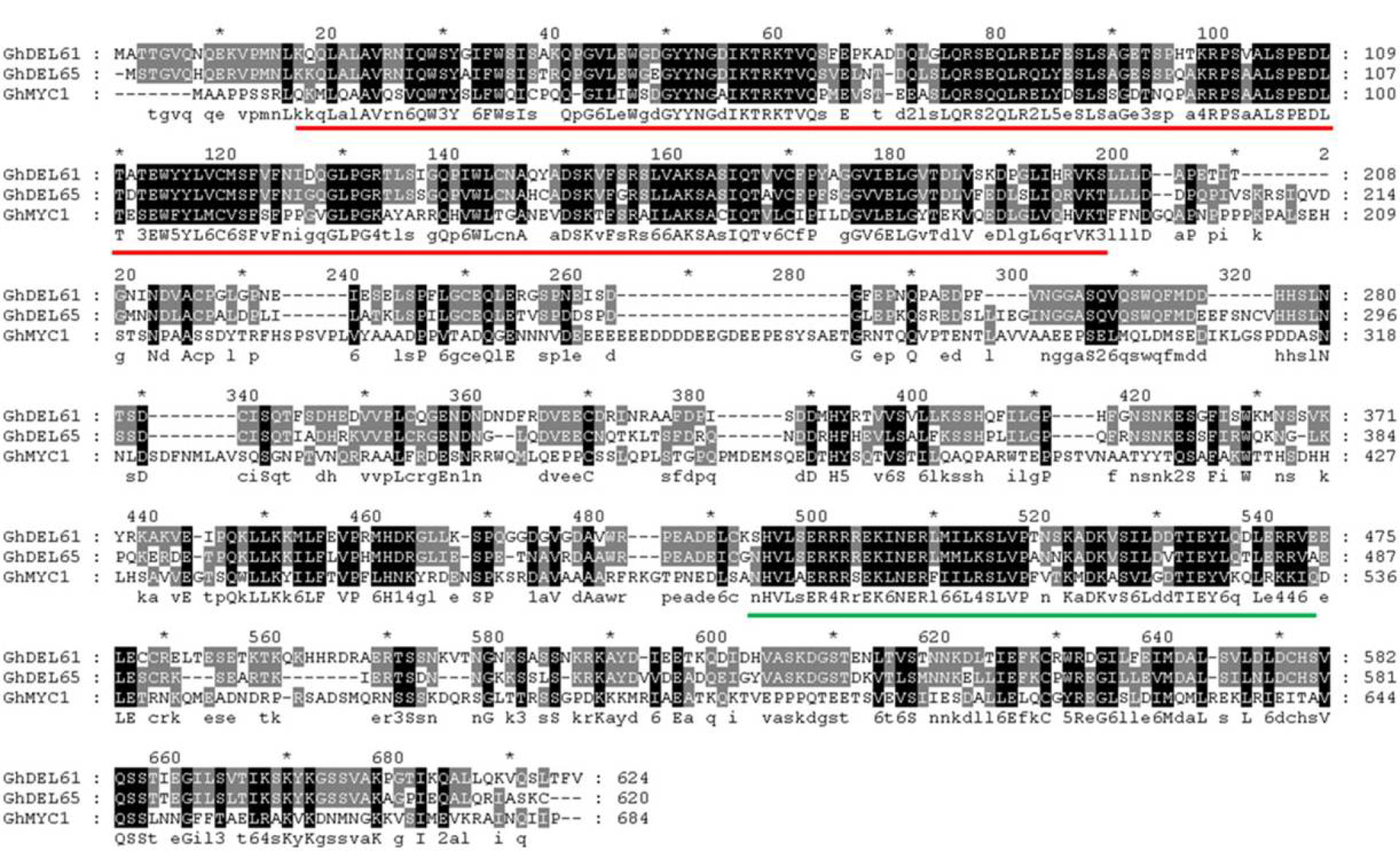

Supplement: S6 Fig — (TIF) [file pone.0116272.s006.tif]

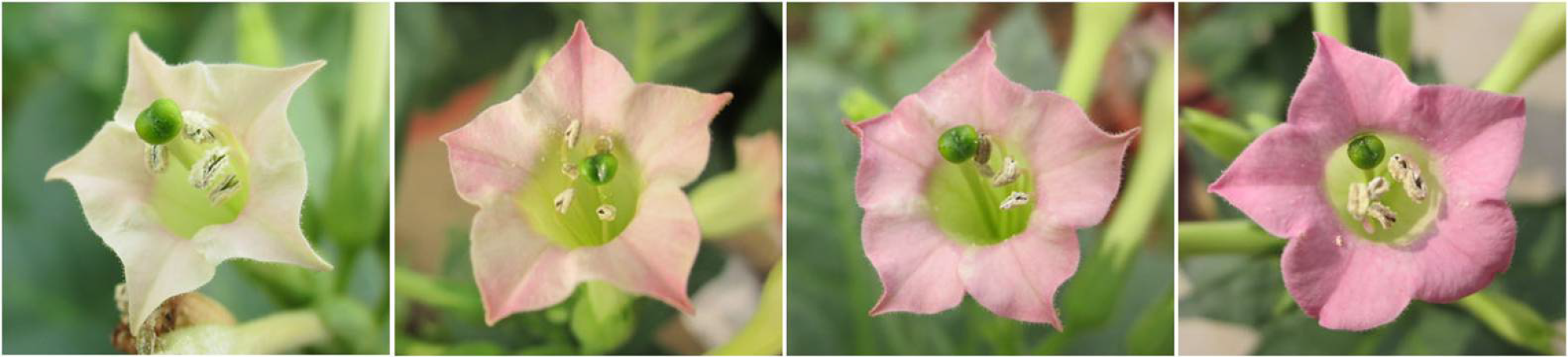

Supplement: S7 Fig — (TIF) [file pone.0116272.s007.tif]
